# Phosphorylation of caspase-8 by RSKs *via* organ-constrained effects controls the sensitivity to TNF-induced death

**DOI:** 10.1038/s41420-024-02024-0

**Published:** 2024-05-24

**Authors:** Peng He, Tingting Ai, Muzhen Qiao, Zhang-Hua Yang, Jiahuai Han

**Affiliations:** 1 Research Unit of Cellular Stress of CAMS, Xiang’an Hospital of Xiamen University, Cancer Research Center of Xiamen University, School of Medicine, Faculty of Medicine and Life Sciences, Xiamen University, Xiamen, 361102 China; 2 State Key Laboratory of Cellular Stress Biology, School of Life Sciences, Faculty of Medicine and Life Sciences, Xiamen University, Xiamen, 361102 China; 3 Department of Gastroenterology, Sir Run Run Shaw Hospital, Zhejiang University School of Medicine, Zhejiang University, Hangzhou, 310012 China; 4 Laboratory Animal Center, Faculty of Medicine and Life Sciences, Xiamen University, Xiamen, 361102 China

**Keywords:** Necroptosis, Stress signalling, Sepsis

## Abstract

Caspase-8 (Casp8) serves as an initiator of apoptosis or a suppressor of necroptosis in context-dependent manner. Members of the p90 RSK family can phosphorylate caspase-8 at threonine-265 (T265), which can inactivate caspase-8 for bypassing caspase-8-mediated blockade of necroptosis and can also decrease caspase-8 level by promoting its degradation. Mutating T265 in caspase-8 to alanine (A) in mice blocked TNF-induced necroptotic cecum damage but resulted in unexpectedly massive injury in the small intestine. Here, we show RSK1, RSK2, and RSK3 redundantly function in caspase-8 phosphorylation, and the duodenum is the most severely affected part of the small intestine when T265 phosphorylation of caspase-8 was prevented. Eliminating caspase-8 phosphorylation resulted in a duodenum-specific increase in basal caspase-8 protein level, which shall be responsible for the increased sensitivity to TNF-induced damage. Apoptosis of intestinal epithelial cells (IECs) was predominant in the duodenum of TNF-treated *Rsk1*^−^^*/*^^−^*Rsk2*^−^^*/*^^−^*Rsk3*^−^^*/*^^−^ and *Casp8*^*T265A/T265A*^ mice, though necroptosis was also observed. The heightened duodenal injury amplified systemic inflammatory responses, as evidenced by the contribution of hematopoietic cells to the sensitization of TNF-induced animal death. Further analysis revealed that hematopoietic and non-hematopoietic cells contributed differentially to cytokine production in response to the increased cell death. Collectively, RSKs emerges as a previously overlooked regulator that, *via* tissue/organ-constrained inactivating caspase-8 and/or downregulating caspase-8 protein level, controls the sensitivity to TNF-induced organ injury and animal death.

## Introduction

Tumor necrosis factor-α (TNF) is a pleiotropic inflammatory cytokine and plays important roles in cell death and inflammatory genes expression [1]. Direct injection of TNF into mice can trigger systemic inflammatory response syndrome (SIRS) and animal death [2–4]. Necroptosis and apoptosis in intestines are known to be crucial for TNF-induced mice death [5–8]. TNF stimulation activates both necroptotic and apoptotic pathways [9, 10]. After TNF-TNF receptor engagement, the TNF receptor recruits adaptors/effectors to form a protein complex termed complex I, which contains TRADD, RIP1 (receptor-interacting protein 1), TRAF2, and cIAP1/2 [9, 10]. Ubiquitinated RIP1 in complex I serves as a platform for activation of NF-κB, the deubiquitylation of RIP1 can switch to form a RIP3-containing complex termed necrosome to initiate necroptosis [9–13]. Pro-caspase-8 (pro-Casp8) in the necrosome is a checkpoint controller [14–17] whose inactivation artificially by caspase inhibitor [18, 19] or naturally by p90 ribosomal s6 kinase (RSK) is often required for the processing of necroptosis [8]. MLKL interacts with RIP3 in the necrosome where it undergoes phosphorylation and then the phosphorylated-MLKL translocates into the plasma membrane to execute necroptosis [20–25]. In the absence of RIP3 expression, complex II (also called Death Inducing Signaling Complex, or DISC) containing RIP1, caspase-8, TRADD, and FADD is formed instead, to initiate caspase cascade and subsequent apoptotic cell death [8, 26].

RSK is a family of highly conserved Ser/Thr kinases, including four members, RSK1, RSK2, RSK3, and a slightly distantly related RSK4, which regulate diverse cellular processes, such as cell growth, cell motility, and cell survival [27, 28]. RSK contains an N-terminal kinase domain (NTKD) sharing homology with the protein kinase A, G, and C family, a conserved linker region, and a C-terminal kinase domain (CTKD) sharing homology with calcium/calmodulin-dependent protein kinase (CAMK) family [27, 29]. All members of the RSK family express across multiple tissues [30]. RSK can directly phosphorylate caspase-8 at threonine-265 (T265, T263 in human) residue, leading to the destabilization of caspase-8 through ubiquitination [31] or inactivation of its protease activity which permits the occurrence of necroptosis [8]. Although the RSK family members could phosphorylate caspase-8 in vitro [8, 31], whether and how the RSK family members contribute to TNF-induced cellular/tissue injuries in vivo is still elusive. It was shown that the N-terminal kinase activity of RSK is required to phosphorylate caspase-8 in TNF-treated cells, but the well-characterized activation mechanism of RSK (e.g., the sequential activation of the C-terminal kinase and the N-terminal kinase of RSK [27, 29]) does not apply to the phosphorylation of caspase-8. The activation of the C-terminal kinase by ERK is not involved in TNF-induced activation of the N-terminal kinase of RSK. Rather, RSK in necroptotic cells is activated by PDK1 through a non-canonical mechanism [8]. Since PDK1 being broadly expressed and constitutively active [32], the regulation of caspase-8 by RSK might represent a homeostatic control mechanism. On the other hand, RSK2-mediated caspase-8 phosphorylation has been reported to be involved in EGF-induced caspase-8 ubiquitination and degradation [31].

In this study, we addressed the role of different RSK family members in TNF responses in vivo. Our data demonstrated that the alterations in the intestinal damage by the T265A mutation of caspase-8 in our previous report are attributed to the elimination of RSK-mediated phosphorylation of T265 in caspase-8. We showed that RSK1, RSK2, and RSK3 function redundantly in phosphorylation of caspase-8. T265 phosphorylation of caspase-8 in TNF-treated mice suppresses caspase-8 activity in cecum which promotes necroptosis. Whereas in duodenum the elimination of T265 phosphorylation of caspase-8 by T265A mutation increases caspase-8 protein level which enhances apoptosis and necroptosis. Thus, through phosphorylation of caspase-8, RSK family members participate in the tissue/organ damage elicited by TNF-induced pathogenic changes in vivo.

## Results

### Triple knockout of *Rsk1, Rsk2*, and *Rsk3* resembles the T265A mutation of caspase-8 in inhibiting cecum damage and promoting small intestine injury upon TNF treatment

Our previous study showed that RSK1 and/or RSK2 can phosphorylate the T265 residue of caspase-8 in a cell type-dependent manner, which is primarily determined by the expression level of RSK1 or RSK2 [8]. T265 phosphorylation inactivates caspase-8 and thereby eliminates caspase-8 mediated inhibition of necroptosis [8]. The effect of T265A mutation of caspase-8 in vivo has organ/tissue-specific effects [8]. Our previous work has demonstrated that it inhibits cecum injury *via* blocking necroptosis, however, it also sensitizes the small intestine to TNF-induced injury by an unknown mechanism [8]. Whether these organ/tissue-restricted opposite effects result solely from the phosphorylation of caspase-8 on T265 by one or more RSKs, or from an unknown function of T265A mutation not linked to the T265 phosphorylation by RSKs, awaits further investigation.

**Fig. 1:**
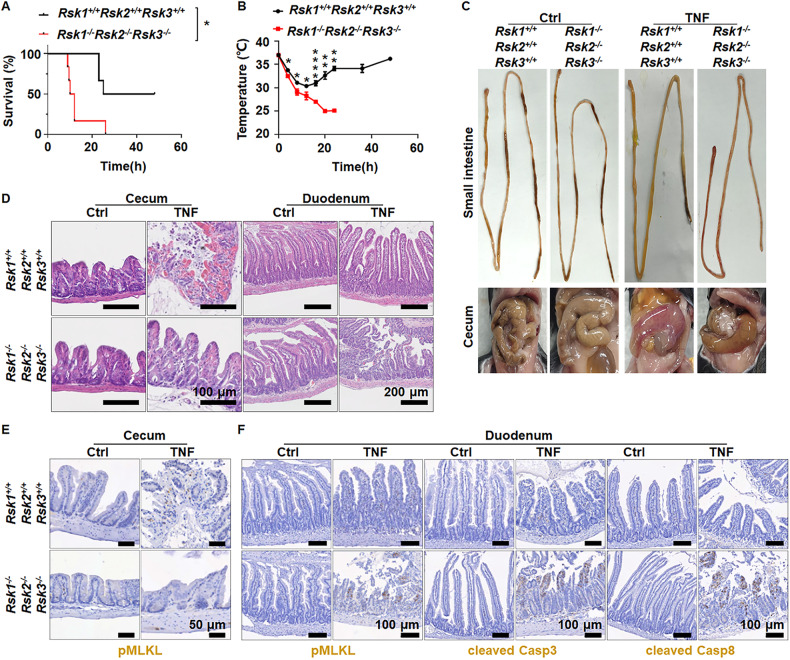
Triple knockout of *Rsk1, Rsk2*, and *Rsk3* enhances TNF-induced mouse death by promoting apoptosis and necroptosis in duodenal epithelial cells. *Rsk1*^*+/+*^*Rsk2*^*+/+*^*Rsk3*^*+/+*^ and *Rsk1*^−^^*/*^^−^*Rsk2*^−^^*/*^^−^*Rsk3*^−^^*/*^^−^ mice were injected intravenously (i.v.) with TNF (0.4 μg/g, *n* = 6), survival curve (**A**) and body temperature (**B**) were recorded at the indicated time. Mouse survival was presented as a Kaplan–Meier plot, and the log-rank test was performed. ns, *p* ≥ 0.05; *p < 0.05; **p < 0.01; ***p < 0.001; ****p < 0.0001. **C** Representative images of the gastrointestinal tract from *Rsk1*^*+/+*^*Rsk2*^*+/+*^*Rsk3*^*+/+*^ or *Rsk1*^−^^*/*^^−^*Rsk2*^−^^*/*^^−^*Rsk3*^−^^*/*^^−^ mice i.v. injected with 0.4 μg/g TNF for 8 h. **D** Representative H&E staining images of pathological changes of cecum and duodenum of *Rsk1*^*+/+*^*Rsk2*^*+/+*^*Rsk3*^*+/+*^ and *Rsk1*^−^^*/*^^−^*Rsk2*^−^^*/*^^−^*Rsk3*^−^^*/*^^−^ mice i.v. injected with TNF (0.4 μg/g) for 8 h. Scale bars as indicated. **E** Representative pMLKL immunochemistry staining images of cecum from *Rsk1*^*+/+*^*Rsk2*^*+/+*^*Rsk3*^*+/+*^ and *Rsk1*^−^^*/*^^−^*Rsk2*^−^^*/*^^−^*Rsk3*^−^^*/*^^−^ mice i.v. injected with TNF (0.4 μg/g) for 8 h. Scale bars as indicated. **F** Representative pMLKL, cleaved Casp3 and cleaved Casp8 immunochemistry staining images of duodenum from *Rsk1*^*+/+*^*Rsk2*^*+/+*^*Rsk3*^*+/+*^ and *Rsk1*^−^^*/*^^−^*Rsk2*^−^^*/*^^−^*Rsk3*^−^^*/*^^−^ mice i.v. injected with TNF (0.4 μg/g) for 8 h. Scale bars as indicated.

**Fig. 2:**
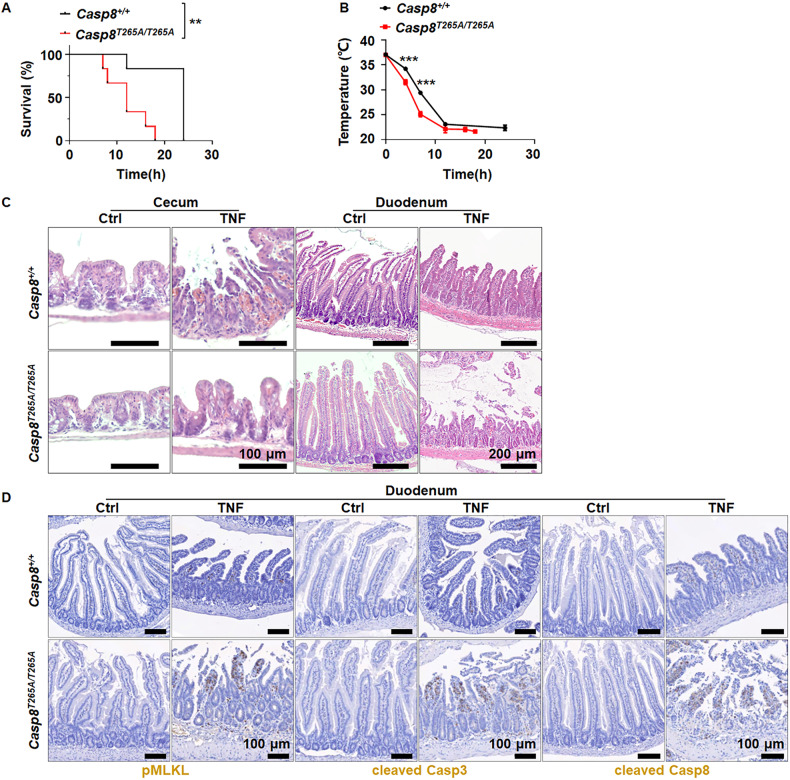
The T265A mutation of caspase-8 enhances TNF-induced mouse death by promoting apoptosis and necroptosis in duodenal epithelial cells. *Casp8*^*+/+*^ and *Casp8*^*T265A/T265A*^ mice were i.v. injected with TNF (0.4 μg/g, *n* = 6), survival curve (**A**), and body temperature (**B**) were recorded at the indicated time. Mouse survival was presented as a Kaplan–Meier plot, and the log-rank test was performed. ns, *p* ≥ 0.05; *p < 0.05; **p < 0.01; ***p < 0.001; ****p < 0.0001. **C** Representative H&E staining images of cecum and duodenum from *Casp8*^*+/+*^ and *Caspase-8*^*T265A/T265A*^ mice i.v. injected with TNF (0.4 μg/g) for 8 h. Scale bars as indicated. **D** Representative images of immunochemistry staining of pMLKL, cleaved Casp3, and cleaved Casp8 in the duodenum of *Casp8*^*+/+*^ and *Casp8*^*T265A/T265A*^ mice i.v. injected with TNF (0.4 μg/g) for 8 h. Scale bars as indicated.

We generated genetic deletion of each RSK family member (Supplementary Fig. S1) and found that none of the single gene deletion in mice showed difference in TNF-induced mice death and cecum injury (Supplementary Fig. S2A–E). Apparently, RSKs are redundant in influencing TNF-induced mice death, as *Rsk1* and *Rsk2* double deletion still did not affect TNF-induced mice death (Supplementary Fig. S2A) and cecum injury (Supplementary Fig. S2D, E), but triple knockout of *Rsk1*, *Rsk2*, and *Rsk3* sensitized mice to TNF-induced death (Fig. 1A, B). Similar to our observations in our previous study in *Casp8*^*T265A/T265A*^ mice [8] (Fig. 2), *Rsk1*^−^^*/*^^−^*Rsk2*^−^^*/*^^−^*Rsk3*^−^^*/*^^−^ mice showed damage in the small intestine but cecum injury was markedly compromised (Fig. 1C). Further analysis revealed that the most severe damage was in the duodenum. Damaged architecture and signs of hemorrhage were observed in *Rsk1*^−^^*/*^^−^*Rsk2*^−^^*/*^^−^*Rsk3*^−^^*/*^^−^ mice and *Casp8*^*T265A/T265A*^ mice but not in WT mice (Figs. 1D, and 2C). On the other hand, noticeable destruction of architecture and hemorrhage was observed in the cecum of WT mice, but not in *Rsk1*^−^^*/*^^−^*Rsk2*^−^^*/*^^−^*Rsk3*^−^^*/*^^−^ mice and *Casp8*^*T265A/T265A*^ mice (Figs. 1D, and 2C). Phosphorylation of MLKL (pMLKL) was easily detected in the cecum of WT but hardly in *Rsk1*^−^^*/*^^−^*Rsk2*^−^^*/*^^−^*Rsk3*^−^^*/*^^−^ mice (Fig. 1E), and pMLKL, cleaved caspase-3 (cleaved Casp3) and cleaved Casp8 were all detected in the duodenum of *Rsk1*^−^^*/*^^−^*Rsk2*^−^^*/*^^−^*Rsk3*^−^^*/*^^−^ and *Casp8*^*T265A/T265A*^ mice but not in WT mice (Figs. 1F and 2D). The triple knockout of *Rsk1*, *Rsk2*, and *Rsk3* did not have a noticeable influence on the responses of the ileum, jejunum, and colon, as well as kidney, liver, lung, pancreas, and spleen upon TNF stimulation in vivo (Supplementary Fig. S3). These findings are consistent with those observed in *Casp8*^*T265A/T265A*^ mice [8] (Supplementary Fig. S4). Based on these results, we conclude that RSK1, RSK2 and RSK3 are redundant in vivo in the phosphorylation of T265 of caspase-8; both the protective effect on cecum damage and the sensitizing effect on duodenum injury caused by T265A mutation of caspase-8 result from blocking RSK-mediated T265 phosphorylation; and T265 phosphorylation in cecum promotes necroptosis but suppresses cell death including apoptosis and necroptosis in the duodenum.

### Both hematopoietic and non-hematopoietic cells participate in the modulation of TNF-induced *Casp8*^*T265A/T265A*^ mice death

**Fig. 3:**
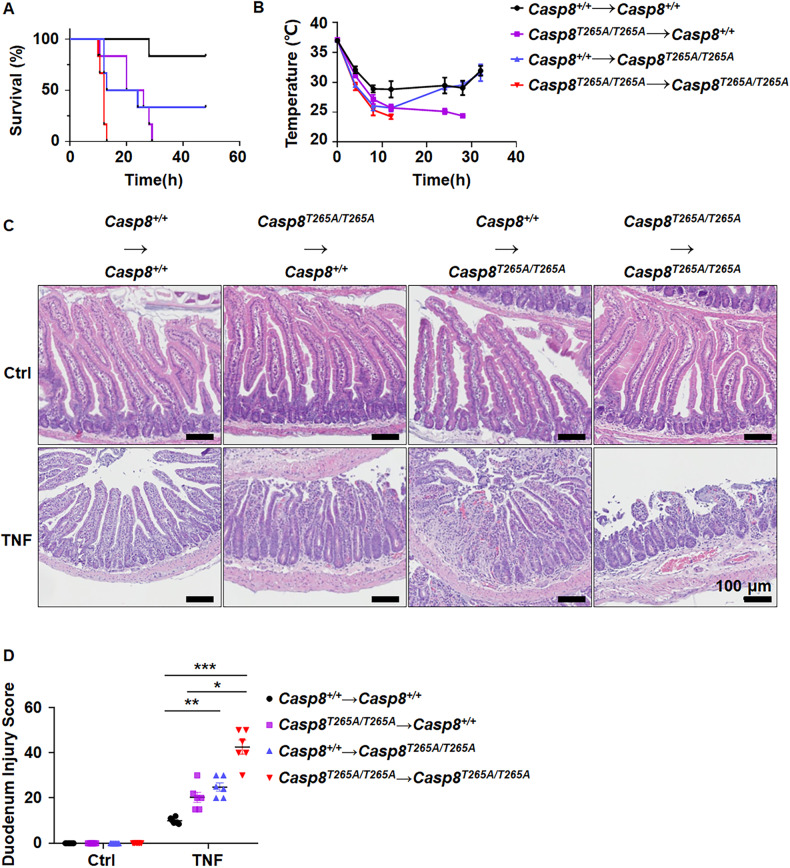
The enhanced TNF responses in *Casp8*^*T265A/T265A*^ mice are attributed by both hematopoietic and non-hematopoietic cells. Bone marrow transplantation was performed and the genotypes of the donors and recipients are indicated. The mice were i.v. injected with TNF (0.2 μg/g, *n* = 6), survival curve (**A**), and body temperature (**B**) were recorded at the indicated time. Mouse survival was presented as a Kaplan–Meier plot, and the log-rank test was performed. **C**, **D** The H&E staining for the duodenum of indicated mice was performed at 8 h after TNF (0.2 μg/g) i.v. injection. Scale bars as indicated (**C**). Tissue injury of indicated mice was scored (*n* = 6 per group) (**D**). ns, *p* ≥ 0.05; *p ＜0.05; **p < 0.01; ***p < 0.001; ****p < 0.0001.

To explore the cell types that participate in the sensitization to TNF-induced death in *Casp8*^*T265A/T265A*^ mice, we conducted bone marrow transplantation using lethally irradiated WT and *Casp8*^*T265A/T265A*^ mice as recipients, and healthy WT or *Casp8*^*T265A/T265A*^ mice as donors. WT mice that received bone marrow from *Casp8*^*T265A/T265A*^ mice exhibited higher sensitivity to TNF (Fig. 3A, B) than those receiving bone marrow transplantation from WT mice, but less sensitive than *Casp8*^*T265A/T265A*^ mice that received bone marrow from *Casp8*^*T265A/T265A*^ mice. Conversely, *Casp8*^*T265A/T265A*^ mice that received bone marrow from WT mice displayed reduced sensitivity to TNF (Fig. 3A, B) in comparison to those receiving bone marrow from *Casp8*^*T265A/T265A*^ mice, but more sensitive than WT mice receiving bone marrow from WT mice. In line with the survival data that after TNF stimulation, WT mice transplanted with *Casp8*^*T265A/T265A*^ bone marrow showed more severe duodenal damage than those transplanted with WT bone marrow, while the *Casp8*^*T265A/T265A*^ mice transplanted with WT bone marrow showed less duodenal damage than those transplanted with *Casp8*^*T265A/T265A*^ bone marrow (Fig. 3C, D). Thus, both non-hematopoietic cells and hematopoietic cells have functions in TNF-induced *Casp8*^*T265A/T265A*^ mice death.

### Preventing RSK-mediated caspase-8 phosphorylation selectively increases caspase-8 in the duodenum and the elevation of caspase-8 protein level correlates with the sensitization of duodenum to TNF-induced injury

**Fig. 4:**
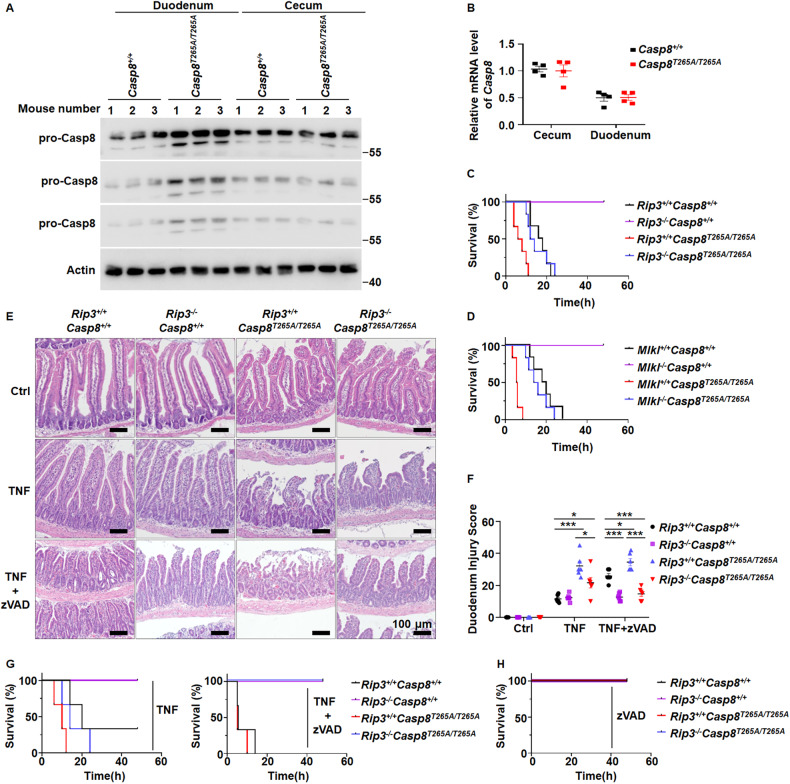
The protein level of pro-caspase-8 in duodenal IECs of *Casp8*^*T265A/T265A*^ mice is selectively increased and blocking apoptosis and necroptosis simultaneously inhibits TNF-induced death of *Casp8*^*T265A/T265A*^ mice. **A** IECs from indicated mice (*n* = 3) were isolated from the cecum or duodenum and Western blotting was performed. pro-Casp8 (1 min, 45 s, and 15 s exposure times were shown) and actin were detected. **B** qRT‒PCR analysis of *Casp8* mRNA levels in IECs from indicated mice (*n* = 4). **C**, **D** Mice with different genotypes as indicated were i.v. injected with TNF (0.4 μg/g, *n* = 6), survival curves were recorded at the indicated time. Mouse survival was presented as a Kaplan–Meier plot, and the log-rank test was performed. **E**, **F** The H&E staining for the duodenum of indicated mice was performed at 8 h after i.v. injection of TNF (0.4 μg/g) or TNF plus intraperitoneal injections (i.p) of zVAD at 15 min before (250 μg per mice) and 1 h after (100 μg per mice) TNF injection (**E**). Scale bars as indicated. Tissue injury of indicated mice was scored (*n* = 6 per group) (**F**). ns, *p* ≥ 0.05; *p＜0.05; **p＜0.01; ***p＜0.001; ****p＜0.0001. **G**, **H** Mice were i.v. injected with TNF (0.4 μg/g), or TNF plus intraperitoneal injections (i.p) of zVAD at 15 min before (250 μg per mice) and 1 h after (100 μg per mice) TNF injection, or zVAD alone. Survival curves were recorded at the indicated time (*n* = 3). Mouse survival was presented as a Kaplan–Meier plot, and the log-rank test was performed.

It was reported that phosphorylation of caspase-8 by RSKs led to caspase-8 ubiquitination and degradation by proteasome [31]. We therefore analyzed caspase-8 level in the cecum and duodenum from wild-type and *Casp8*^*T265A/T265A*^ mice. Western blotting revealed that the caspase-8 protein level but not its mRNA level in the duodenal IECs of *Casp8*^*T265A/T265A*^ mice was obviously higher than that in wild-type mice (Fig. 4A, B). In contrast, there was no difference of caspase-8 expression between the cecal IECs from wild-type and *Casp8*^*T265A/T265A*^ mice (Fig. 4A). These data explain well the increased sensitivity of duodenum but not cecum to TNF-induced damage in *Rsk1*^−^^*/*^^−^*Rsk2*^−^^*/*^^−^*Rsk3*^−^^*/*^^−^ and *Casp8*^*T265A/T265A*^ mice.

Since both pMLKL and cleaved Casp8/cleaved Casp3 were detected in the duodenum of *Casp8*^*T265A/T265A*^ mice after TNF treatment (Fig. 2D), the increase expression of caspase-8 appeared to enhance both apoptosis and necroptosis. We crossed *Rip3*^−^^*/*^^−^ or *Mlkl*^−^^*/*^^−^ mice with *Casp8*^*T265A/T265A*^ mice and found that both *Rip3*^−^^*/*^^−^ and *Mlkl*^−^^*/*^^−^ indeed could reduce the sensitivity of *Casp8*^*T265A/T265A*^ mouse to TNF-induced death, but the effect was very modest (Fig. 4C, D). A similar effect was observed when duodenal damages were analyzed (Fig. 4E) and duodenal injury was scored (Fig. 4F). Thus, necroptosis still takes part in the damage of the small intestine in TNF-treated *Casp8*^*T265A/T265A*^ mice. However, duodenal damage of *Casp8*^*T265A/T265A*^ mice should result more from apoptosis than necroptosis due to the detection of massive cleaved Casp8 and cleaved Casp3 (Fig. 2D). To explore the role of caspase-3, we generated *Casp3*^−^^*/*^^−^*Casp8*^*T265A/T265A*^ mice. As reported by others, deletion of *Casp3* in mice causes developmental abnormalities [33] and *Casp3*^−^^*/*^^−^ mice are already very susceptible to TNF-induced death (Supplementary Fig. S5). *Casp3*^−^^*/*^^−^*Casp8*^*T265A/T265A*^ mice also showed more susceptibility to TNF-induced death than *Casp8*^*T265A/T265A*^ mice (Supplementary Fig. S5), but we cannot draw conclusions regarding the requirement of caspase-3 based on these data.

We then tested the inhibition of caspases by the pan-caspase inhibitor zVAD in mice. Compared to TNF treatment, TNF+zVAD induced more severe damage in the duodenum of WT mice and similar damage in the duodenum of *Casp8*^*T265A/T265A*^ mice (Fig. 4E, F). Of note, applying zVAD nearly completely blocked TNF-induced duodenum damage in *Rip3*^−^^*/*^^−^*Casp8*^*T265A/T265A*^ mice (Fig. 4E, F). More importantly, as shown in Fig. 4G, zVAD had minimal effect on *Casp8*^*T265A/T265A*^ mice but applying zVAD on *Rip3*^−^^*/*^^−^*Casp8*^*T265A/T265A*^ mice prevented mice death. zVAD by itself did not have any influence on the viability of mice in the absence of TNF treatment (Fig. 4H). Thus, apoptosis and necroptosis together elicit the hypersensitivity of *Casp8*^*T265A/T265A*^ mice to TNF-induced death.

### Impairment of RSK-mediated caspase-8 phosphorylation upsurges in vivo inflammation

**Fig. 5:**
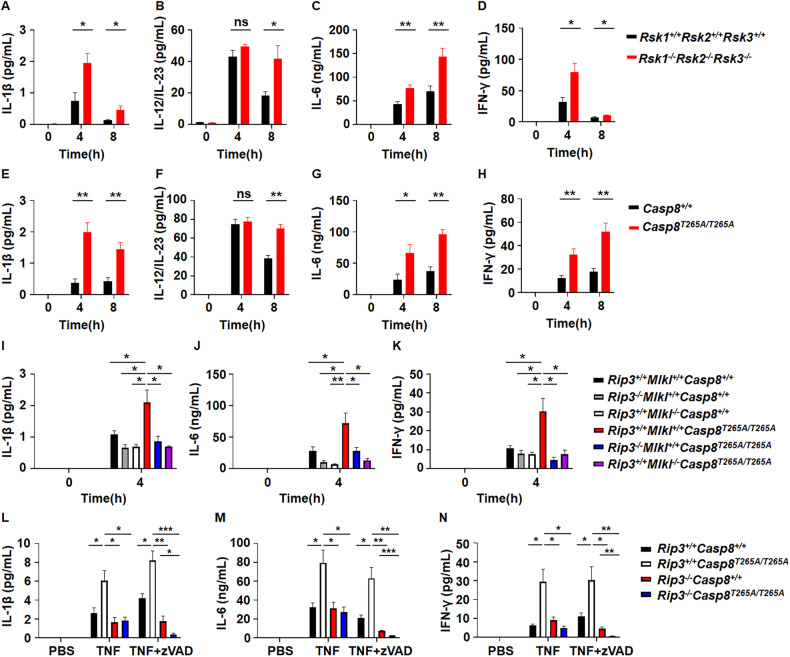
TNF or TNF + zVAD induces more cytokines production in *Rsk1*^−^^*/*^^−^*Rsk2*^−^^*/*^^−^*Rsk3*^−^^*/*^^−^ or *Casp8*^*T265A/T265A*^ mice than that in WT mice. **A**–**D** *Rsk1*^*+/+*^*Rsk2*^*+/+*^*Rsk3*^*+/+*^ and *Rsk1*^−^^*/*^^−^*Rsk2*^−^^*/*^^−^*Rsk3*^−^^*/*^^−^ mice were i.v. injected with TNF (0.4 μg/g). At the indicated time points, mouse serum was collected for ELISA analysis of IL-1β (**A**), IL-12/IL-23 (**B**), IL-6 (**C**), and IFN-γ (**D**) (*n* = 6 per group at each time point). **E**–**H** *Casp8*^*+/+*^ and *Casp8* ^*T265A/T265A*^ mice were i.v. injected with TNF (0.4 μg/g). At the indicated time points, mouse serum was collected for ELISA analysis of IL-1β (**E**), IL-12/IL-23 (**F**), IL-6 (**G**), and IFN-γ (**H**) (*n* = 4 per group at each time point). Mice of indicated genotypes were i.v. injected with TNF (0.4 μg/g). 4 h after TNF injection, mouse serum was collected for ELISA analysis of IL-1β (**I**), IL-6 (**J**), and IFN-γ (**K**) (*n* = 4 per group at each time point). Mice of indicated genotypes were i.v. injected with TNF (0.4 μg/g) plus i.p injections of zVAD at 15 min before (250 μg per mouse) and 1 h after (100 μg per mouse) TNF injection. 4 h after TNF injection, mouse serum was collected for ELISA analyzation of IL-1β (**L**), IL-6 (**M**), and IFN-γ (**N**) (*n* = 4 per group at each time point). ns, *p* ≥ 0.05; *p < 0.05; **p < 0.01; ***p < 0.001; ****p < 0.0001.

TNF is a pro-inflammatory cytokine that can stimulate the expression of other inflammatory cytokines in cultured cells [10]. We measured several cytokines in the blood of TNF-stimulated *Rsk1*^−^^*/*^^−^*Rsk2*^−^^*/*^^−^*Rsk3*^−^^*/*^^−^, *Casp8*^*T265A/T265A*^, and WT mice. Increases of IL-1β, IL-12/23, IL-6, and IFN-γ were observed and the level of increases of these cytokines was higher in *Rsk1*^−^^*/*^^−^*Rsk2*^−^^*/*^^−^*Rsk3*^−^^*/*^^−^ and *Casp8*^*T265A/T265A*^ mice compared to WT mice (Fig. 5A–H).

We treated BMDMs derived from *Casp8*^*T265A/T265A*^ or WT mice with TNF and found that TNF did not induce much IL-1β, IFN-γ, and IL-6 production as well as NF-κB and MAP kinase activation in BMDMs (Supplementary Fig. S6A–D). Direct stimulation of macrophages with TNF may not be the cause of the increased cytokine expression in *Casp8*^*T265A/T265A*^ mice.

We then analyzed the levels of the cytokines when cell death was blocked. The mice and treatments are described in Fig. 4C–G. Blocking necroptosis by deletion of *Rip3* or *Mlkl* reduced cytokine induction in *Casp8*^*T265A/T265A*^ mice (Fig. 5I–K), and additional inhibition of apoptosis by zVAD in *Rip3*^−^^*/*^^−^*Casp8*^*T265A/T265A*^ mice almost completely eliminated cytokine production (Fig. 5L–N). Thus, the enhanced cytokine production by the T265A mutation of caspase-8 should be mainly elicited by increased cell death.

Dead cells shall release DAMPs, which activate cellular responses *via* the Myd88 pathway [34]. We tested the deletion of *Myd88* in *Casp8*^*T265A/T265A*^ mice and found a significant reduction in the sensitivity to TNF-induced mice death (Supplementary Fig. S7), supporting the idea that cell death is an intermediate step in animal death. Furthermore, when treating BMDMs derived from *Rsk1*^−^^*/*^^−^*Rsk2*^−^^*/*^^−^*Rsk3*^−^^*/*^^−^, *Casp8*^*T265A/T265A*^, or WT mice with TNF, we observed no induction of death or caspase-3/7 activation (Supplementary Fig. S8A–D). We also tested whether *Zbp1*, *Gsdme*, or *Gsdmd* participates in the sensitization of *Casp8*^*T265A/T265A*^ mice to TNF-induced mice death and excluded their involvement (Supplementary Fig. S9A–C).

**Fig. 6:**
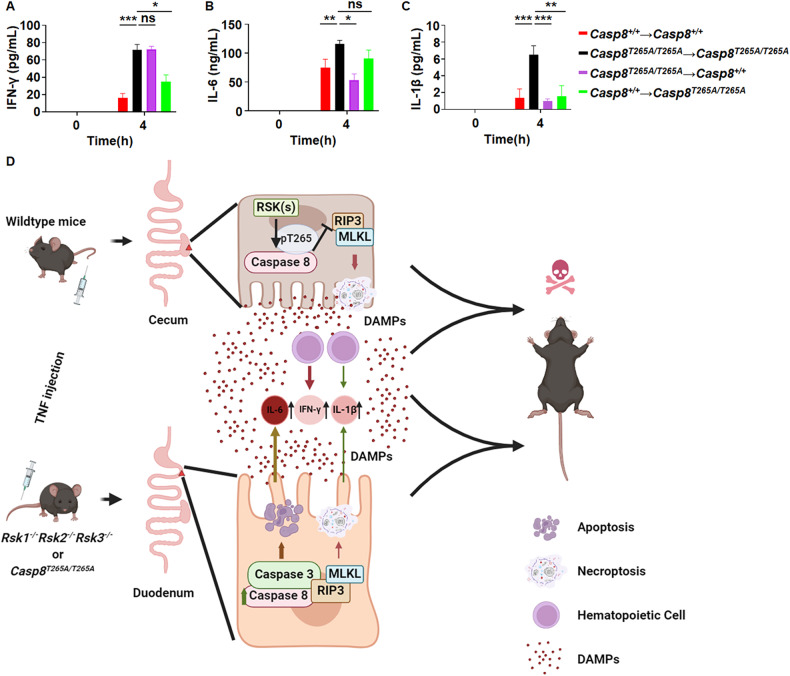
Hematopoietic and non-hematopoietic cells differentially mediate the enhancement of different cytokine expressions in TNF-treated *Casp8*^*T265A/T265A*^ mice. Mice with bone marrow transplantation as indicated were i.v. injected with TNF (0.2 μg/g). 4 h after TNF injection, mouse serum was collected for ELISA analysis of IFN-γ (**A**), IL-6 (**B**), and IL-1β (**C**) (*n* = 4 per group at each time point). ns, *p* ≥ 0.05; *p < 0.05; **p < 0.01; ***p < 0.001; ****p < 0.0001. **D** Schematic illustration of the role of RSKs-caspase-8 axis in TNF responses in vivo. In WT mice, RIP3-MLKL-mediated necroptosis makes the cecum the most sensitive organ to TNF-induced injury, and cecum damage initiates animal death. The elimination of caspase-8 phosphorylation in *Rsk1*^−^^*/*^^−^*Rsk2*^−^^*/*^^−^*Rsk3*^−^^*/*^^−^ or *Casp*^*T265A/T265A*^ mice sensitizes the mice to TNF-induced death. With a dose of TNF that cannot induce death in WT mice, the death of duodenal IECs in these mutation mice can be induced by TNF, ultimately leading to animal death. The underlying cause of this phenomenon is most likely to be the selective increase of caspase-8 protein level in the duodenum, which results from eliminated RSKs-mediated phosphorylation of caspase-8. As a consequence, the initial damage organ in *Rsk1*^−^^*/*^^−^*Rsk2*^−^^*/*^^−^*Rsk3*^−^^*/*^^−^ or *Casp*^*T265A/T265A*^ mice switches from cecum to duodenum. Apoptosis, necroptosis to a less extent, is responsible for the duodenal damages. The increased IFN-γ secretion is primarily contributed by hematopoietic cells, whereas IL-6 secretion is by non-hematopoietic cells, and both hematopoietic and non-hematopoietic cells are involved in the secretion of IL-1β. These cytokines are most likely feedback to IECs in the duodenum or cecum to orchestrate TNF-induced damage.

Since the effects of the T265 phosphorylation of caspase-8 by RSKs occur in both hematopoietic and non-hematopoietic cells, we analyzed blood cytokines in bone marrow transplanted mice described in Fig. 3. The increase of IFN-γ production by T265A mutation of caspase-8 was primarily contributed by hematopoietic cells, because WT mice transplanted with *Casp8*^*T265A/T265A*^ bone marrow showed a similar level of IFN-γ as *Casp8*^*T265A/T265A*^ mice transplanted with *Casp8*^*T265A/T265A*^ bone marrow, and *Casp8*^*T265A/T265A*^ mice transplanted with WT bone marrow showed a less level of IFN-γ compared with *Casp8*^*T265A/T265A*^ mice transplanted with *Casp8*^*T265A/T265A*^ bone marrow (Fig. 6A). In contrast, non-hematopoietic cells were the major contributor to IL-6 secretion, since WT mice transplanted with *Casp8*^*T265A/T265A*^ bone marrow or *Casp8*^*T265A/T265A*^ mice transplanted with WT bone marrow did not exhibit a significant influence on their blood IL-6 levels (Fig. 6B). As for the secretion of IL-1β, mutation in both hematopoietic and non-hematopoietic cells is required for its secretion (Fig. 6C). Thus, the increase of different cytokines by T265A mutation of caspase-8 in response to TNF is mediated by different cells.

## Discussion

Phosphorylation of caspase-8 at T265 residue by RSK is an intrinsic mechanism for bypassing the caspase-8 checkpoint of necroptosis [8]. While the regulation mechanism of this checkpoint has been well documented, an unexpected phenotype was observed that the *Casp8*^*T265A/T265A*^ mouse is not more resistant but rather more sensitive to TNF-induced death. Even more striking is that this phenomenon is associated with a switch of the primary damage organ from the cecum to the small intestine [8]. The studies presented in the manuscript excluded the possibility that this unexpected phenotype does not result from the compromise of caspase-8 phosphorylation by RSKs. A published study has shown that phosphorylation of caspase-8 by RSK can lead to caspase-8 degradation [31] and we did observe increased caspase-8 protein in the duodenum but not the cecum in *Casp8*^*T265A/T265A*^ mice (Fig. 4A). Based on the data presented in this and published studies, we proposed a model to interpret the role of RSKs in TNF-induced mouse death (Fig. 6D). In cecum, necroptosis is the predominant cell death pathway induced by TNF [5, 8]. Caspase-8 is a checkpoint controller that prevents the occurrence of necroptosis [14–17]. Inactivation of caspase-8 by RSKs-mediated phosphorylation permits necroptosis [8]. Whereas in the small intestine, especially in the duodenum, caspase-8 level is regulated by RSK-mediated phosphorylation as elimination of caspase-8 phosphorylation led to increased caspase-8 protein level in *Casp8*^*T265A/T265A*^ mice (Fig. 4A). Massive damage in the duodenum of *Rsk1*^−^^*/*^^−^*Rsk2*^−^^*/*^^−^*Rsk3*^−^^*/*^^−^ or *Casp8*^*T265A/T265A*^ mice was induced by TNF even at lower dose is due to duodenum restricted increase of caspase-8 protein. The RSK-mediated control of cell death pathways exhibits organ/tissue-specific effects, and the death suppression by RSKs could be essential in maintaining the homeostasis of the small intestine, as the elimination of T265 phosphorylation in caspase-8 converts duodenum to the most sensitive organ to TNF-induced damage.

Mechanistically, the sensitization to TNF-induced death in *Rsk1*^−^^*/*^^−^*Rsk2*^−^^*/*^^−^*Rsk3*^−^^*/*^^−^ or *Casp8*^*T265A/T265A*^ mice by increased caspase-8 protein could be attributed to many possible causes. Some factors may control the sensitivity and others may determine the threshold for the responses. Here in this study, we revealed that the organ selective elevation of caspase-8 protein level should be a major cause of the increased sensitivity of duodenum to TNF-induced damage. Duodenum may inherently be able to sense low concentrations of TNF, but without the removal of RSK-mediated suppression and degradation of caspase-8, the death pathway cannot be initiated. Our previous work demonstrated that the recruitment of RSKs to necrosomes led to the inactivation of caspase-8 [8]. However, in the case of organ-specific properties, RSK’s suppression of caspase-8 may be mediated by its constitutive basal activity. Although caspase-8’s suppressive effect on necroptosis is obvious in the cecum, it appears to be involved in both necroptosis and apoptosis in the duodenum. In the latter case, massively formed caspase-8 containing complex II may still recruit some RIP3 and caspase-8, and caspase-8-T265A cannot completely cut RIP1/RIP3. Thus, despite the majority of caspase-8 is matured, necroptosis can still be detected in the duodenum.

RSKs are multifunctional kinases that participate in various biological processes, including the regulation of pro-inflammatory response [35]. RSKs phosphorylate IκBα at Ser32 residue and p65 at Ser536 residue to activate NF-κB [36, 37]. RSKs also regulate the secretion of cytokines, such as IL-6 [38], and participate in the expression of cyclooxygenase 2 (COX2) to increase prostaglandin production to increase inflammatory response [39]. Through the phosphorylation of caspase-8, RSKs regulate TNF-induced cell death, thereby negatively modulating inflammatory responses.

The role of caspase-8 in cell death has been well established [9]. It promotes apoptosis, inhibits necroptosis, and regulates inflammasomes [40]. Additionally, *caspase-8* deficiency in dendritic cells was shown to be linked to uncontrolled TLR activation, characterized by massive cytokine induction, such as IL-12, and IL-6 [41]. In contrast, caspase-8 promotes TLR4-induced NF-κB activation in mouse B cells independent of its protease enzymatic activity [42], whereas the production of inflammatory cytokines (TNF, IL-6, IL-12) upon *Yersinia* infection relies on the protease enzymatic activity of caspase-8 [43]. An interferon-inducible viral restriction factor N4BP1 was found to be downstream of caspase-8 in inflammatory response [44, 45]. Here in this study, we observed an increase of blood cytokines in *Casp8*^*T265A/T265A*^ and *Rsk1*^−^^*/*^^−^*Rsk2*^−^^*/*^^−^*Rsk3*^−^^*/*^^−^ mice upon TNF treatment. The increases of different cytokines were mediated by different types of cells and should result primarily from their responses to the DAMPs released from the dead cells.

In short, this study demonstrates that RSK1, RSK2, and RSK3 function redundantly in suppressing caspase-8 in vivo. While suppression of caspase-8 activity through RSKs phosphorylation in necrosomes promotes necroptosis, promoting caspase-8 degradation by RSKs phosphorylation also inhibits apoptosis on other occasions. Given the conserved and fundamental functions of RSKs in individual growth and homeostasis, the regulation of caspase-8 by RSKs sheds more light on the interplay among pathways of growth and death in inflammatory responses. This study implies that the classic RSK pathway could be a target for developing innovative therapeutic treatment for systemic inflammatory diseases.

## Materials and methods

### Cell culture

BMDMs (Bone Marrow-Derived Macrophage, mouse male or female, sex-matched) were generated as previously described [46]. Briefly, differentiating bone marrow progenitors from the tibia and femur for 7 days in Dulbecco’s modified Eagle’s medium (DMEM, 12800-082, Gibco, USA) supplemented with 10% (v/v) fetal bovine serum (FBS, SH30071.03, HyClone, USA) and 30% (v/v) L929-conditioned medium. All cells were grown at 37 °C in a 5% CO_2_ incubator. Reagents used in this study: TNF (PMC3015, Thermo Fisher, USA), SM164 (A8815, APExBIO, USA), zVAD (627610, Calbiochem, Germany), and Nec-1 (480065, EMD Chemicals, Germany). All cell strains were well established and frequently checked by monitoring morphology and functionalities.

### Cell death assay

Cell death was analyzed using CellTiter-Glo Luminescent Cell Viability Assays kit (G7571, Promega, USA). The Luminescent Cell Viability Assays were performed according to the manufacturer’s instructions [8]. In brief, 1.0 × 10^5^ cells were seeded in 96-well plates with white wall. After treatment, an equal volume of CellTiter-Glo reagent was added to the cell culture medium, which had been equilibrated to room temperature for 30 min. Cells were shaken at 220 rpm for 15 min at room temperature. Luminescent recording was performed with Spark 20 M microplate reader (Tecan, Switzerland).

### Caspase-3/7

Caspase-3/7 activity was determined by using a Caspase-Glo 3/7 assay kit (G8092, Promega, USA) according to the manufacturer’s instructions [8]. In brief, 1.0 × 10^5^ cells were seeded in 96-well plates with white wall. After treatment, an equal volume of Caspase-Glo 8 reagent was added to the cell culture medium, which had been equilibrated to room temperature for 30 min. Cells were shaken at 220 rpm for 15 min at room temperature. Luminescent recording was performed with Spark 20 M microplate reader (Tecan, Switzerland).

### Intestinal epithelial cells (IECs) harvest

IECs were extracted from indicated genotype mice as described previously [47]. Briefly, the intestines were cut longitudinally, divided into 1 cm pieces, and incubated in ice-cold PBS containing 6 mM EDTA (pH 8.0) for 1 h. The remaining intestines were then suspended in PBS and shaken violently 15 times to release epithelium cells from the basement membrane. IECs were centrifuged at 400 × *g* for 3 min at 4 °C. After washing once with ice-cold PBS at 4 °C, the IECs were lysed in 1× SDS sample buffer for Western blotting.

### Immunoblot analysis

Immunoblot was performed as previously described [8]. Antibodies used included: pro-caspase-8 (4790, Cell Signaling, USA), p38α (9228, Cell Signaling, USA), p-p38 (9211, Cell Signaling, USA), ERK (9107, Cell Signaling, USA), p-ERK (9101, Cell Signaling, USA), JNK (9252, Cell Signaling, USA), p-JNK (9251, Cell Signaling, USA), IκB-α (9242, Cell Signaling, USA), Actin (Santa Cruz Biotechnology, SC-47778), and GAPDH (AC002, ABclonal, China). The full-length uncropped original western blots were provided in the supplementary file.

### Mice

*Rsk*^−^^*/*^^−^ was home generated by CRSIPR/Cas9-mediated gene editing in the mouse zygote. gRNA: 5′-GCCGGGTGACCTTGCGTACC-3′ and 5′- TGACGTGAACCACCCGTTCG-3′ for *Rsk1* knockout. gRNA: 5′-GGAGAGCCCTTCCGACAGCG-3′ for *Rsk2* knockout. gRNA: 5′-TGGAGCGCGACTTCTTGCGC-3′ and 5′-GAGAGCACAGGCTCTAGCCT-3′ for *Rsk3* knockout. gRNA: 5′-ACCTGTGGCCAGATGGCGTG-3′ and 5′-GCTAAACGTGAAGCAGTAAC-3′ for *Rsk4* knockout. The disruption of the target gene was determined by the sequencing of gene loci and by the immunoblotting of cell lysates with antibodies. *Mlkl*^−^^*/*^^−^ mice were generated by transcription activator-like effector nucleases (TALENs)-mediated gene-disruption method in a C57BL/6 background, as described previously [46]. *Casp8*^*T265A/T265A*^ [8] and *Casp3*^−^^*/*^^−^ [48] mice were generated by CRISPR/Cas9 and haploid embryonic stem cell systems [49]. *Gsdmd*^−^^*/*^^−^ [50], *Zbp1*^−^^*/*^^−^ [51], and *Gsdme*^*−/−*^ [52] mice were generated by co-microinjection of in vitro-translated Cas9 mRNA and gRNA into the C57BL/6 zygotes. *Rip3*^*−/−*^ mice were a kind gift from Dr. Vishva Dixit (Genentech, South San Francisco, CA) as we described previously [53]. Additional information is provided upon request. All the mice were housed and fed in a specific pathogen-free facility a 12 h light-dark cycle (light time: 8 a.m. to 8 p.m.) at the Xiamen University Laboratory Animal Center, and the room temperature was at 22–24 °C and room humidity was at 50–70%. All the mice were back-crossed to the C57BL/6 J background for at least 6 generations. Unless stated otherwise, both male and female mice (8–12 weeks old) were used randomly in this study, and the experimental/control animals were co-housed.

### Mouse SIRS model

Mouse SIRS model was conducted as previously reported [3, 5, 7, 8]. Mice were housed in a specific pathogen-free environment. All experiments were conducted in compliance with the regulations of Xiamen University. Six- to eight-week-old male mice (average weight approximately 20 g) were injected intravenously (i.v.) with Mouse TNF (CF09, Novoprotein, China) as indicated concentration, which was diluted in endotoxin-free PBS. zVAD (627610, Calbiochem, Germany) treatment as previously reported [3]. Briefly, zVAD was given 15 min before (250 μg per mouse) and 1 h after (100 μg per mouse) TNF injection through intraperitoneally (i.p). Rectal body temperature was recorded with an electric thermometer (ALC-ET03, ALCbio, Shanghai, China) was measured as indicated in the figure legends. Mice were sacrificed at an indicated time or when body temperature was below 23.6 °C [8]. The investigator was blinded to allocation when the mice were injected with TNF and when mice deaths were counted.

### Bone marrow transplantation

Bone marrow transplantation was carried out as previously reported [7]. Briefly, Bone marrow was harvested from donor mouse (6 weeks) femurs and tibias using 27 G syringe Needles and flushed with PBS with penicillin (100 IU/ml), and streptomycin (100 g/ml) into tubes. The cell pellet was spun down at 1000 × *g*. The supernatant was aspirated and the pellet was hemolyzed with hemolytic buffer (C3702, Beyotime, China) for 5 min. The cells were then spun down again and the supernatant was aspirated; then they were resuspended, counted, and diluted to 1 × 10^7^ cells/mL in PBS for use. Recipient mice (6 weeks) were exposed to 8 Gy lethal X-ray irradiation (RS2000Pro, RAD SOURCE, USA), and 4 h letter transplanted with 5 × 10^6^ cells each mouse by tail vein injection. After 8-weeks recovery, the mouse was used in the TNF injection experiment.

### Serum sample collection

For the serum samples, blood was collected from the retro-orbital vein of the mouse, and was coagulated at 37 °C for 30 min. The coagulated blood samples were then centrifuged at a speed of 2000 × *g* for 20 min. The supernatant was then collected and centrifuged at a speed of 5000 × *g* for another 5 min, and the supernatant was collected carefully as the serum samples. All the centrifuge was taken at 4 °C, and the serum was aliquoted and stored at −80 °C ultra-low temperature freezer for future analysis.

### ELISA

Serum samples were collected as mentioned above, and the concentrations of cytokines (IL-6, IFN-γ, IL-12/23, and IL-1β) in serums were measured following the manufacturer’s protocol. Absorbance Microplate Reader (ELX800, BioTek) was used to quantify the value of ELISA reactions. ELISA Kit used in this study were Mouse IL-6 ELISA (88-7064-88, Thermo, USA), Mouse IFN gamma ELISA (88-7314-88, Thermo, USA), Mouse IL-12/23 ELISA (88-7120-88, Invitrogen, USA), Mouse IL-1β (88-7013-77, Thermo, USA).

### Histology

Histology was performed as previously described [8, 54]. Mice were euthanized with carbon dioxide, and tissues or organs were collected immediately and fixed in 4% paraformaldehyde for 24 h for paraffin sections. The fixed tissue samples were dehydrated in ethanol and dewaxed in xylene solution, and finally embedded in paraffin blocks. The paraffin samples were sectioned in a thickness of 5 µm for future analysis. Tissue samples were processed and then stained based on the routine protocol of hematoxylin and eosin (H&E) (hematoxylin: CTS-1096, MXB Biotechnologies, China; eosin: ZLI-9613, ZSGB-BIO, China). The degree of cecum damage was assessed by a method described previously [55]. Then the sum of the average scores of four fields per sample was calculated as the cecum damage score.

For periodic acid-Schiff (PAS, 87007, Thermo, USA) staining methods [56], the kidney tissue sections were stained by 1% periodic acid for 15 min and then washed with ddwater twice. The tissue samples were then stained using Schiff reagent for 15 min, washed, and stained subsequently with hematoxylin for 2 min.

Immunohistochemistry (IHC) analysis was performed as previously described [54]. Briefly, the intestinal tissue sections were put into the slide in the antigen retrieval buffer (10 mM sodium citrate, pH6.0) (W302600, Sigma, Germany) in a pressure cooker, and make sure the whole tissue was embedded in the buffer. Put the pressure cooker on an electromagnetic oven, and heated it under 1800 W. After the cooker began to vent, start timing for 1.5 min and then let the cooker cool to room temperature naturally. Then prepared for blocking and incubating with anti-p-MLKL (S345) antibody (1:1500) (ab196436, Abcam, USA) or anti-cleaved Casp8 (9429, CST, USA) (1:200) and cleaved Casp3 (9661, CST, USA) (1:200), which were diluted in 2.5% horse serum (S-2012-50, Vector laboratories, USA) with chilled PBS at 4 °C overnight, followed by antibody detection with a Horse Anti-Mouse/Rabbit ImmPRESS kit (MP-7500, vector laboratories, USA). Sections were then incubated with diluted streptavidin-peroxidase HRP at room temperature with a staining kit (SK-4105, Vector laboratories, USA), following the manufacturer’s instructions. The sections were then stained with hematoxylin for 2 min.

Representative images were captured and processed using identical settings in the Zeiss AxioScan7 at Xiamen University.

### RNA isolation and quantitative PCR

RNA isolation and quantitative PCR were performed as previously described [46]. Total RNA was extracted using RNAiso Plus reagent (D9109, TaKaRa, Japan), and 3 μg of total RNA was reverse-transcribed into complementary DNA (cDNA) using Hifair® lll lst Strand cDNA SynthesisSuperMix for qPCR (gDNA digester plus) (11141ES10, YEASEN, China). Real-time PCR was performed on a LightCycler 480 System (Roche, Germany) using ChamQ Universal SYBR qPCR Master Mix (Q711-02, Vazyme, China). Data analysis was performed using the 2^−^^△△Ct^ method after normalization to the *ActB* internal control. The primers for mouse *Casp8* are: forward: 5′-ACAAACCTCGGGGATACTGTC-3′; reverse: 5′-AGTGCAGTCGTCGTAAGATACTA-3′. The primers for mouse *ActB* are: forward: 5′-TCCAGCCTTCCTTCTTGGGT-3′; reverse: 5′-GCACTGTGTTGGCATAGAGGT-3′.

### Statistical analysis

Statistical analysis was performed with GraphPad Prism (Version 8.02) and SPSS (version: 27.0). Unpaired two-tailed Student’s *t*-test was used to compare differences between indicated groups and *p*-value was calculated except the cecum damage and kidney damage between two genotypes was analyzed with a Wilcoxon signed-rank test. Mice survival was presented as a Kaplan-Meyer plot and analyzed with a log-rank (Mantel-Cox) Test. Data are expressed as means ± SEM. Differences in compared groups were considered statistically significantly different with *P* values: ns: *p* ≥ 0.05; ^∗^*p* < 0.05; ^∗∗^*p* < 0.01; ^∗∗∗^*p* < 0.001; ^∗∗∗∗^*p* < 0.0001. All images presented in the figures are representative of a minimum of three independent experiments.

### Supplementary information

Supplementary figures

Original Data-Uncropped blots.

## Data Availability

The datasets generated during and/or analyzed during the current study are available from the corresponding author on reasonable request.
