# Supplementary material for: Phosphorylation of caspase-8 by RSKs via organ-constrained effects controls the sensitivity to TNF-induced death
Source: Cell Death Discov. 2024 May 24;10:255. doi: 10.1038/s41420-024-02024-0 (PMC11126741; doi:10.1038/s41420-024-02024-0)
Supplement: Supplementary file 1 — Supplementary figures [file 41420_2024_2024_MOESM1_ESM.pdf]

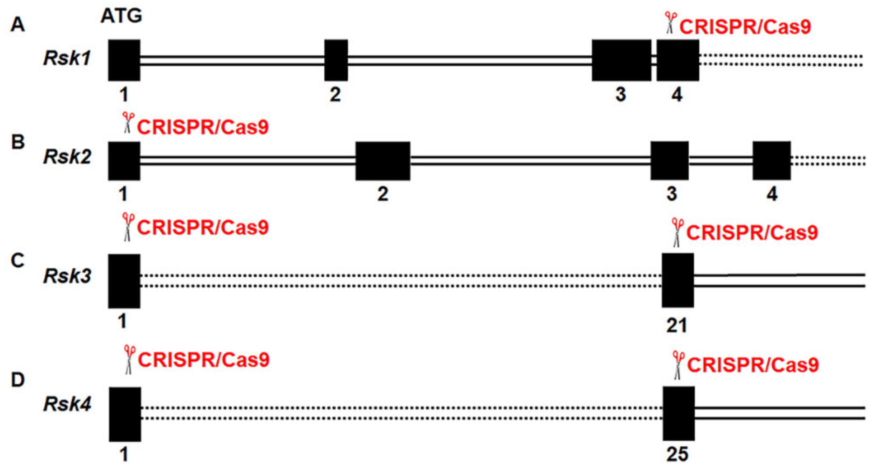

Supplementary Fig S1. Generation of *Rsks* knockout mice by CRISPR/Cas9

Schematics for the CRISPR/Cas9 target exons of the four *Rsks* alleles (**A to D**).

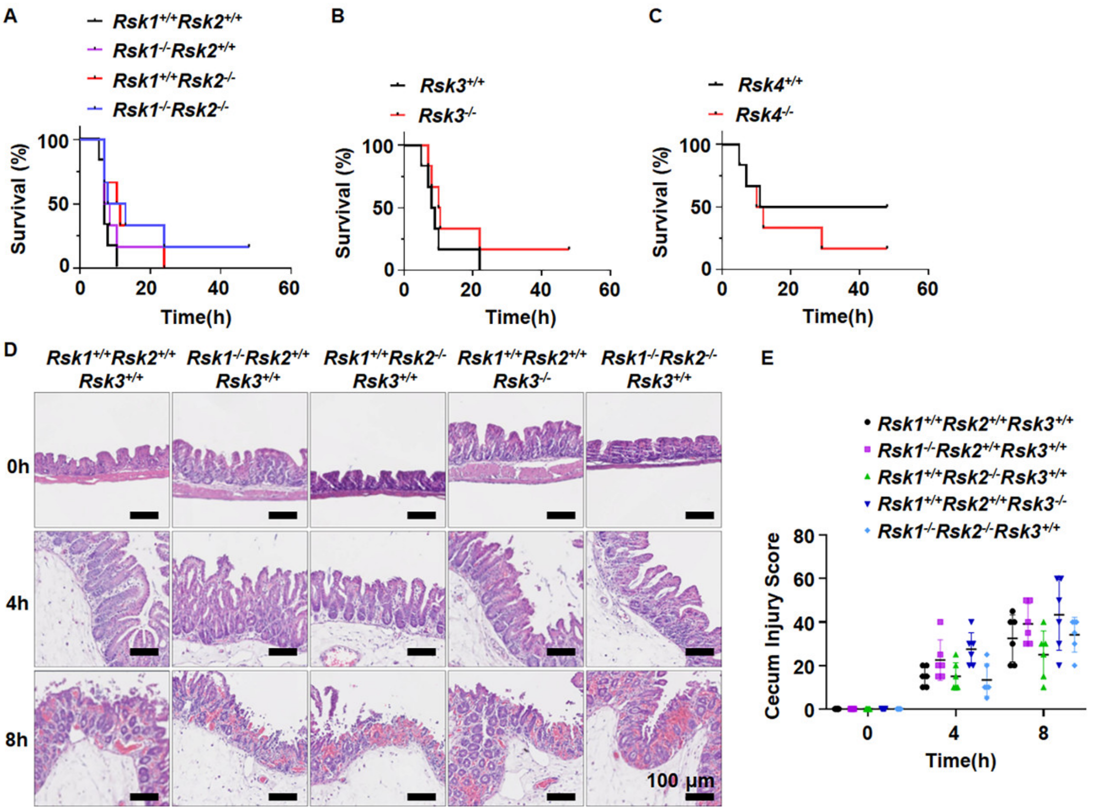

Supplementary Fig S2. Genetic deletion of *Rsk1*, *Rsk2, Rsk3* or *Rsk4* and double deletion of *Rsk1 and Rsk2* did not influence TNF-induced mouse death

(A to C) WT, *Rsk1*$^{-/-}$, *Rsk2*$^{-/-}$, *Rsk1*$^{-/-}$*Rsk2*$^{-/-}$ **(A)**, *Rsk3*$^{-/-}$ **(B)** and *Rsk4*$^{-/-}$ **(C)** mice were i.v. injected with TNF (0.4 μg/g, n=6), the survival curve was recorded at indicated time. Mouse survival was presented as a Kaplan-Meier plot, and the log-rank test was performed.

(D to E) WT, *Rsk1*$^{-/-}$, *Rsk2*$^{-/-}$, *Rsk1*$^{-/-}$*Rsk2*$^{-/-}$, *Rsk3*$^{-/-}$ and *Rsk4*$^{-/-}$ mice were i.v. injected with TNF (0.4 μg/g), and cecum samples were collected at the indicated time points. Representative H&E staining images of cecum are shown **(D)**, and tissue injury was scored (n=6 per group) **(E)**.

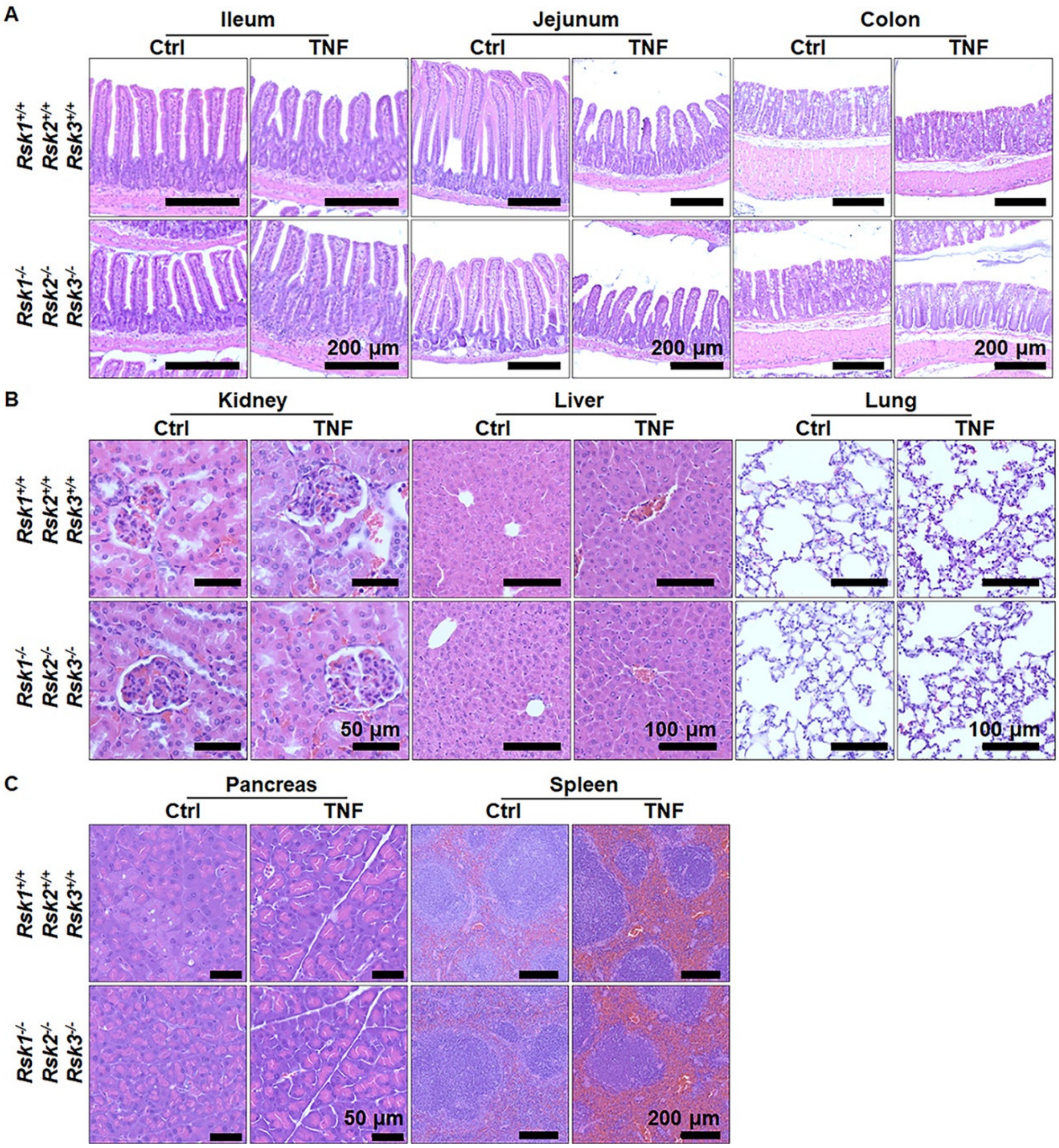

Supplementary Fig S3. Triple knockout of *Rsk1*, *Rsk2*, and *Rsk3* does not affect TNF-induced tissue damage of ileum, jejunum, colon, kidney, liver, lung, pancreas, and spleen

(A) Representative H&E staining images of ileum, jejunum, and colon from $Rsk1^{+/+}Rsk2^{+/+}Rsk3^{+/+}$ or $Rsk1^{-/-}Rsk2^{-/-}Rsk3^{-/-}$ mice were collected at 8 hours after TNF (0.4 µg/g) i.v. injection. Scale bars as indicated.

(B) Representative H&E staining images of kidney, liver, and lung from $Rsk1^{+/+}Rsk2^{+/+}Rsk3^{+/+}$ or $Rsk1^{-/-}Rsk2^{-/-}Rsk3^{-/-}$ mice were collected at 8 hours after

TNF (0.4 μg/g) i.v. injection. Scale bars as indicated.

(C) Representative H&E staining images of pancreas, and spleen from *Rsk1*$^{+/+}$*Rsk2*$^{+/+}$*Rsk3*$^{+/+}$ or *Rsk1*$^{-/-}$*Rsk2*$^{-/-}$*Rsk3*$^{-/-}$ mice were collected at 8 hours after TNF (0.4 μg/g) i.v. injection. Scale bars as indicated.

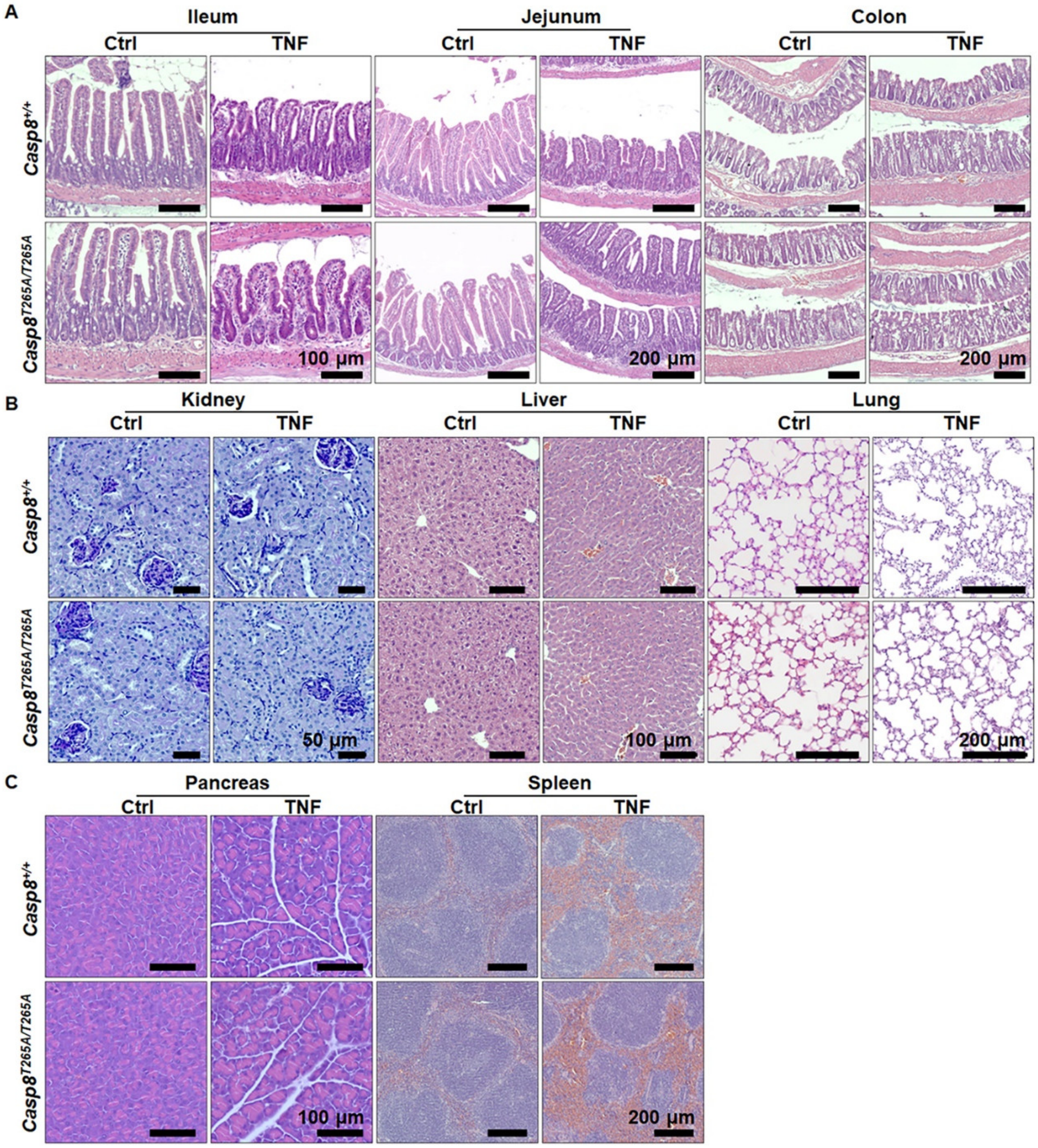

Supplementary Fig S4. The T265A mutation of caspase-8 does not affect TNF-induced tissue damage of ileum, jejunum, colon, kidney, liver, lung, pancreas, and spleen

(A) Representative H&E staining images of ileum, jejunum, and colon from *Casp8*$^{+/+}$ and *Casp8*$^{T265A/T265A}$ mice were collected at 8 hours after TNF (0.4 μg/g) i.v. injection. Scale bars as indicated.

(B) Representative H&E staining images of kidney, liver, and lung from *Casp8*$^{+/+}$ and

Casp8$^{T265A/T265A}$ mice were collected at 8 hours after TNF (0.4 μg/g) i.v. injection. Scale bars as indicated.

(C) Representative H&E staining images of pancreas, and spleen from *Casp8*$^{+/+}$ and *Casp8*$^{T265A/T265A}$ mice were collected at 8 hours after TNF (0.4 μg/g) i.v. injection. Scale bars as indicated.

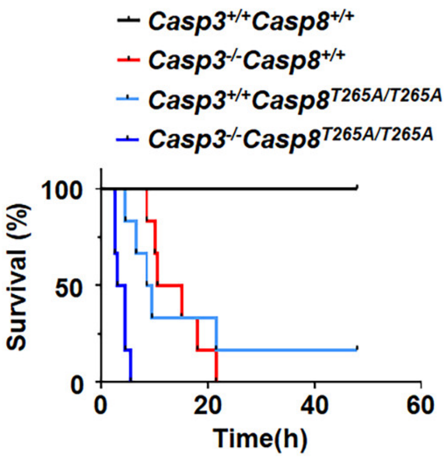

Supplementary Fig S5. Knockout of *Casp3* sensitizes mice to TNF-induced death

WT, *Casp8*$^{T265A/T265A}$, *Casp3*$^{-/-}$, and *Casp3*$^{-/-}$*Casp8*$^{T265A/T265A}$ mice were i.v. injected with TNF (0.2 μg/g, n=6) survival curve was recorded at indicated time. Mouse survival was presented as a Kaplan-Meier plot, and the log-rank test was performed.

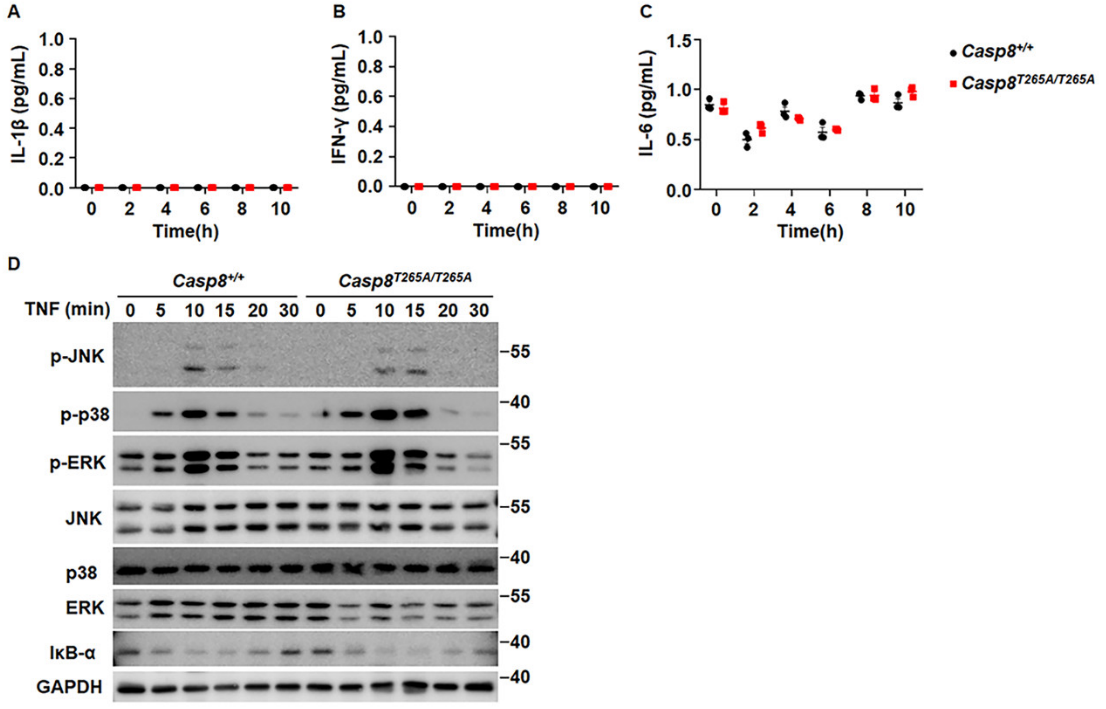

Supplementary Fig S6. The T265A mutation of caspase-8 does not influence TNF-induced inflammatory responses in BMDM

(A to C) BMDMs derived from WT (*Casp8*$^{+/+}$) or *Casp8*$^{T265A/T265A}$ mice were treated with TNF (30 ng/mL). IL-1β **(A)**, IFN-γ **(B)**, and IL-6 **(C)** in culture supernatants were analyzed at the time points as indicated. n = 3 mice per genotype.

(D) The BMDMs were treated as in **(A to C)**. Phosphorylated JNK (p-JNK), p-p38, p-ERK, JNK, p38, ERK, IκB-α, and GADPH were analyzed at the time points as indicated by Western blotting.

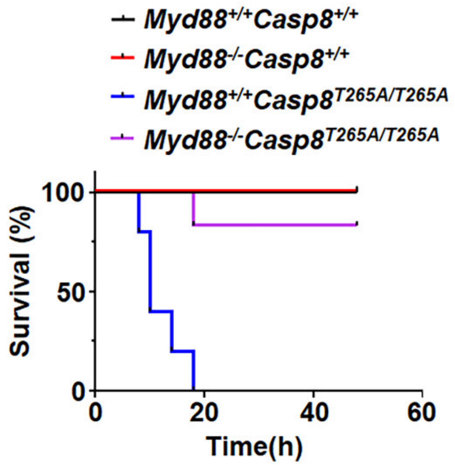

Supplementary Fig S7. Knockout of *Myd88* reduces the sensitivity of *Casp8$^{T265A/T265A}$* mice to TNF-induced death

WT (*Myd88$^{+/+}$Casp8$^{+/+}$*), *Myd88$^{+/+}$Casp8$^{T265A/T265A}$*, *Myd88$^{-/-}$Casp8$^{+/+}$*, and *Myd88$^{-/-}$Casp8$^{T265A/T265A}$* mice were i.v. injected with TNF (0.2 μg/g, n=6). The survival curve was recorded at indicated time. Mouse survival was presented as a Kaplan-Meier plot, and the log-rank test was performed.

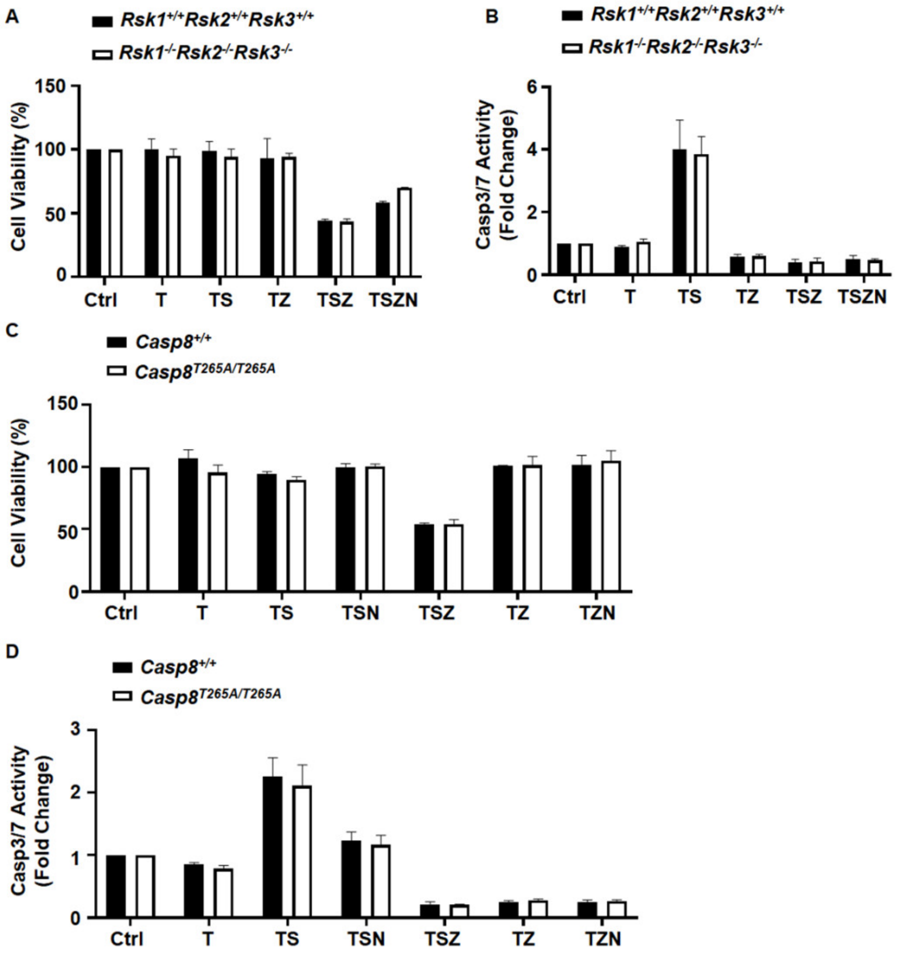

Supplementary Fig S8. Neither triple knockout of *Rsk1*, *Rsk2*, and *Rsk3* nor the T265A mutation of *Casp8* affects the cell death of and caspase 3 activity in BMDMs stimulated with TNF (T), SM164 (S), zVAD (Z), Nec-1 (N) or their combinations

(A and B) Cell viability and Casp3/7 activities were measured in *Rsk1*$^{+/+}$*Rsk2*$^{+/+}$*Rsk3*$^{+/+}$ or *Rsk1*$^{-/-}$*Rsk2*$^{-/-}$*Rsk3*$^{-/-}$ BMDM cells treated as indicated for 24h.

(C and D) Cell viability and Casp3/7 activities were measured in *Casp8*$^{+/+}$ *and* *Casp*$^{T265A/T265A}$ BMDM cells treated as indicated for 24h. TNF (T, 30 ng/mL), SM164 (a Smac-mimetics, S, 100 nM), zVAD (Z, 20 μM), and Nec-1 (N, 30 μM).

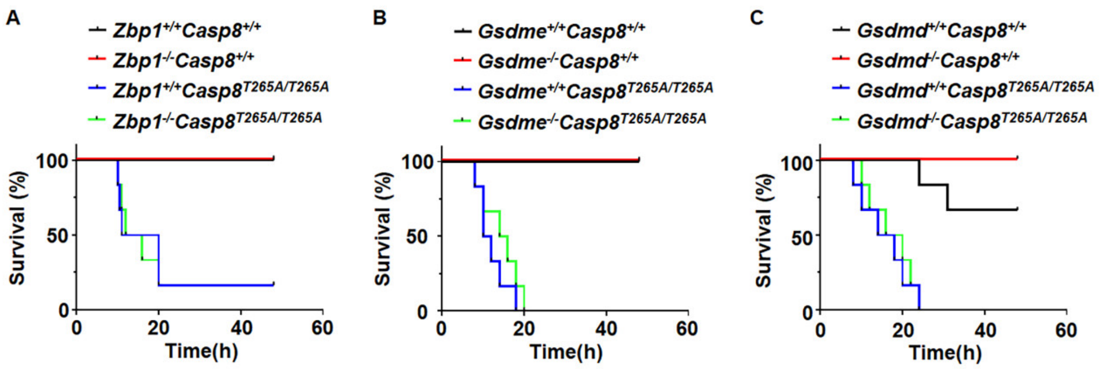

Supplementary Fig S9. Neither *Zbp1*, *Gsdme,* nor *Gsdmd* knockout influences TNF-induced death of *Casp8*$^{T265A/T265A}$ mice

(A to C) WT (*Zbp1*$^{+/+}$*Casp8*$^{+/+}$), *Zbp1*$^{+/+}$*Casp8*$^{T265A/T265A}$, *Zbp1*$^{-/-}$*Casp8*$^{+/+}$, *Zbp1*$^{-/-}$*Casp8*$^{T265A/T265A}$ **(A)**, WT (*Gsdme*$^{+/+}$*Casp8*$^{+/+}$), *Gsdme*$^{+/+}$*Casp8*$^{T265A/T265A}$, *Gsdme*$^{-/-}$*Casp8*$^{+/+}$, *Gsdme*$^{-/-}$*Casp8*$^{T265A/T265A}$ **(B)**, and WT (*Gsdmd*$^{+/+}$*Casp8*$^{+/+}$), *Gsdmd*$^{+/+}$*Casp8*$^{T265A/T265A}$, *Gsdmd*$^{-/-}$ *Casp8*$^{+/+}$, *Gsdmd*$^{-/-}$*Casp8*$^{T265A/T265A}$ **(C)** mice were i.v. injected with TNF (0.2 μg/g, n=6). The survival curve was recorded at indicated time. Mouse survival was presented as a Kaplan-Meier plot, and the log-rank test was performed.
