# Supplementary material for: Phosphorylation of caspase-8 by RSKs via organ-constrained effects controls the sensitivity to TNF-induced death
Source: Cell Death Discov. 2024 May 24;10:255. doi: 10.1038/s41420-024-02024-0 (PMC11126741; doi:10.1038/s41420-024-02024-0)

Figure 4a

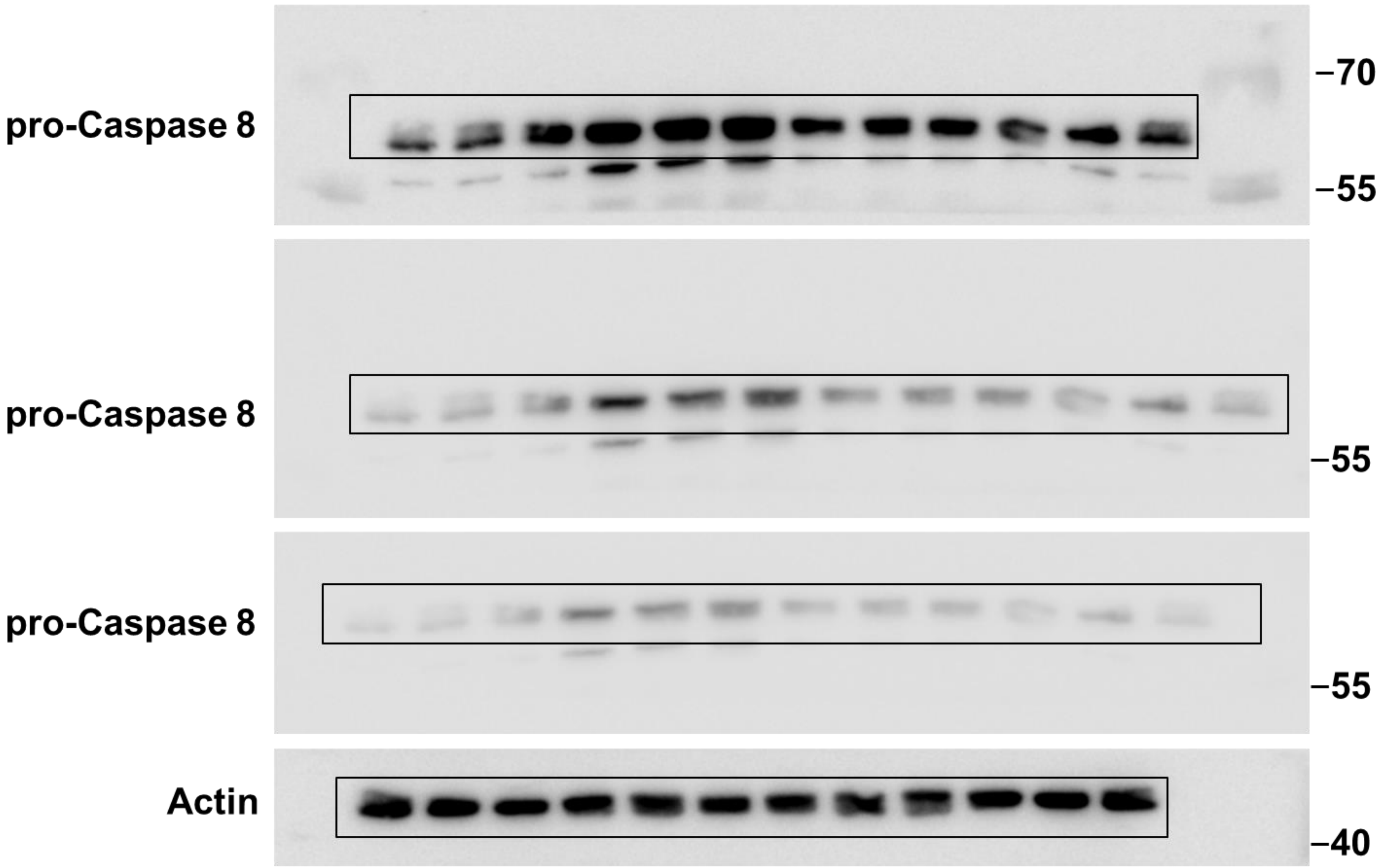

Figure S6d

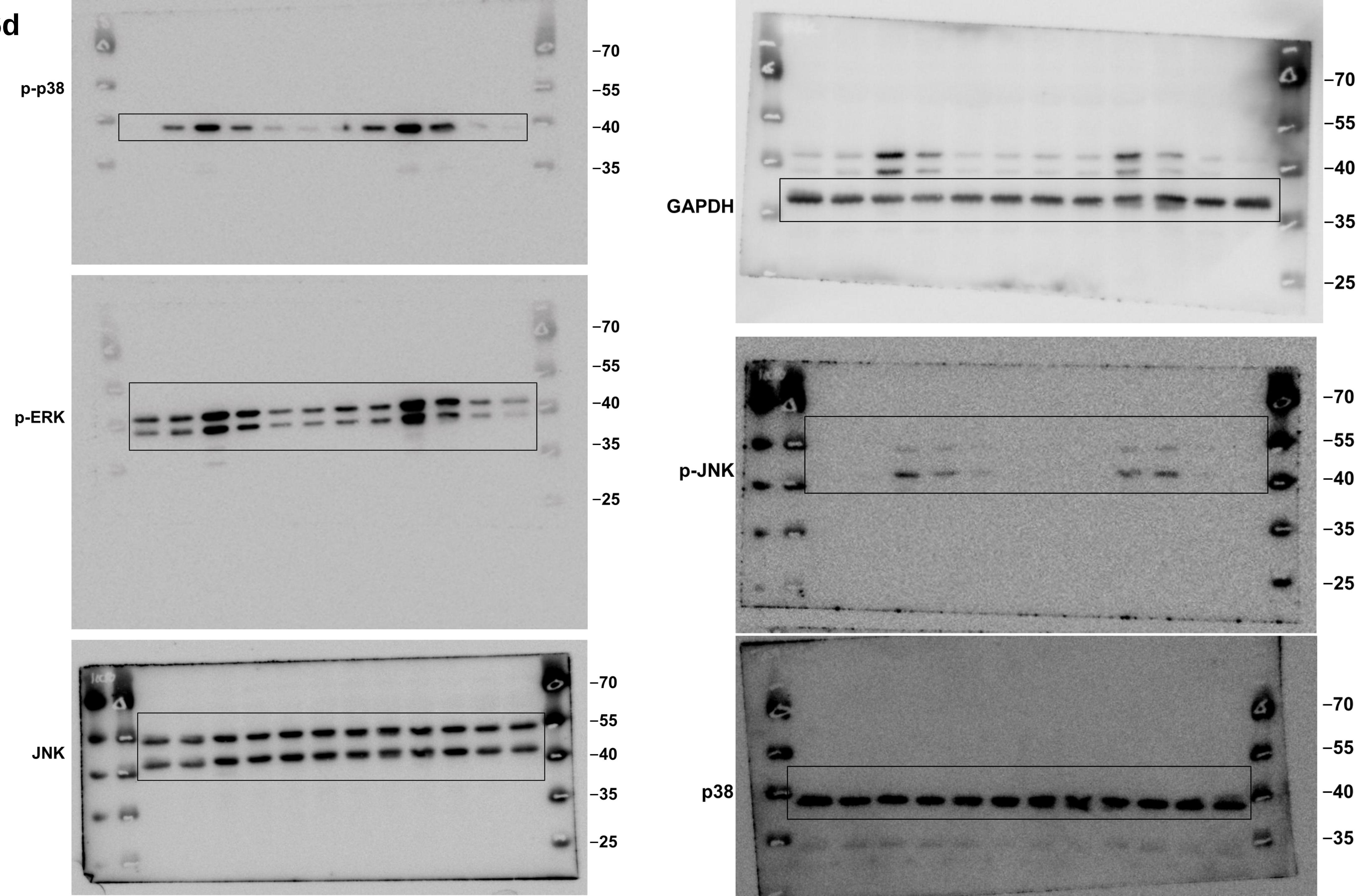

p-p38
–70
–55
–40
–35
p-ERK
–70
–55
–40
–35
–25
JNK
–70
–55
–40
–35
–25
GAPDH
–70
–55
–40
–35
–25
p-JNK
–70
–55
–40
–35
–25
p38
–70
–55
–40
–35

Figure S6d

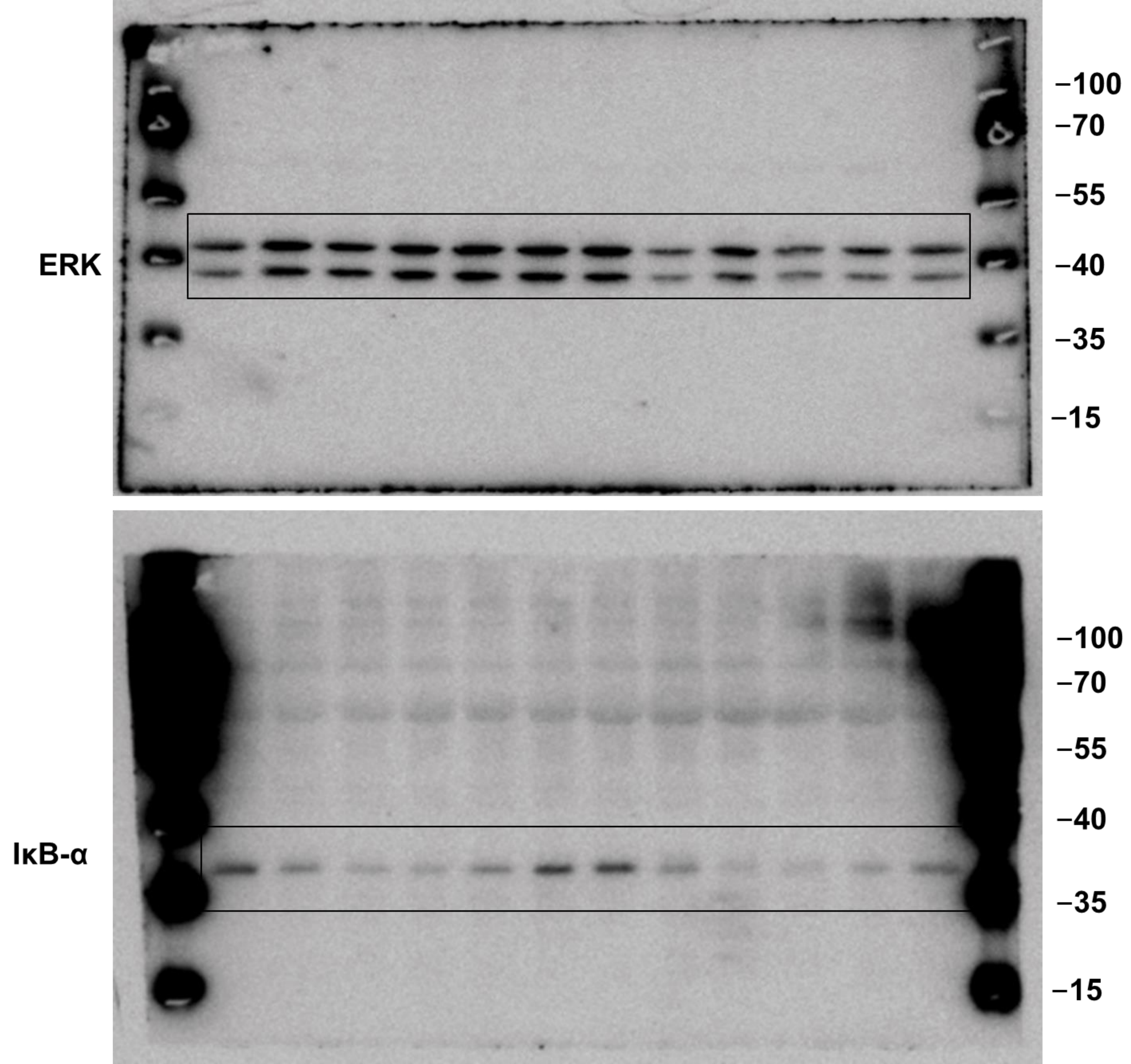

Supplement: Supplementary file 2 — Original Data-Uncropped blots. [file 41420_2024_2024_MOESM2_ESM.pdf]
